# Short-Term Effects of Early Menopause on Adiposity, Fatty Acids Profile and Insulin Sensitivity of a Swine Model of Female Obesity

**DOI:** 10.3390/biology9090284

**Published:** 2020-09-11

**Authors:** Ana Heras-Molina, José Luis Pesantez-Pacheco, Marta Vazquez-Gomez, Consolacion Garcia-Contreras, Susana Astiz, Beatriz Isabel, Antonio Gonzalez-Bulnes

**Affiliations:** 1 Instituto Nacional de Investigación y Tecnología Agraria y Alimentaria (INIA), Ctra. De La Coruña Km. 7,5., 28040 Madrid, Spain; delasheras.ana@inia.es (A.H.-M.); jose.pesantez@ucuenca.edu.ec (J.L.P.-P.); congarcon@gmail.com (C.G.-C.); astiz.susana@inia.es (S.A.); 2 School of Veterinary Medicine and Zootechnics, Faculty of Agricultural Sciences, University of Cuenca, Avda. Doce de Octubre, Cuenca 010220, Ecuador; 3 Faculty of Veterinary Sciences, Universidad Complutense de Madrid, 28040 Madrid, Spain; martavazgomez@gmail.com (M.V.-G.); bisabelr@ucm.es (B.I.); 4 Facultat de Veterinària, Universitat Autònoma de Barcelona. Edifici V, Trav. dels Turons, 08193 Bellaterra, Spain

**Keywords:** models, fatty-acids, insulin-resistance, menopause, obesity, swine

## Abstract

Menopause strongly increases incidence and consequences of obesity and non-communicable diseases in women, with recent research suggesting a very early onset of changes in lipid accumulation, dyslipidemia, and insulin resistance. However, there is a lack of adequate preclinical models for its study. The present trial evaluated the usefulness of an alternative method to surgical ovariectomy, the administration of two doses of a GnRH analogue-protein conjugate (Vacsincel^®^), for inducing ovarian inactivity in sows used as preclinical models of obesity and menopause. All the sows treated with the compound developed ovarian stoppage after the second dose and, when exposed to obesogenic diets during the following three months, showed changes in the patterns of fat deposition, in the fatty acids profiles at the different tissues and in the plasma concentrations of fructosamine, urea, β-hydroxibutirate, and haptoglobin when compared to obese fed with the same diet but maintaining ovarian activity. Altogether, these results indicate that menopause early augments the deleterious effects induced by overfeeding and obesity on metabolic traits, paving the way for future research on physiopathology of these conditions and possible therapeutic targets using the swine model.

## 1. Introduction

Obesity is currently declared a global pandemic according to WHO (https://www.who.int/health-topics/obesity#tab=tab_1 and http://www.who.int/mediacentre/factsheets/fs311/en/index.html) because its worldwide incidence has nearly tripled since 1975 and currently at least 4 million people are dying each year as a direct result of the condition. Moreover, overweight and lifestyle are major risk factors for non-communicable diseases (NCDs) such as metabolic syndrome, cardiovascular disease (CVD), type-2 diabetes (T2D), musculoskeletal disorders, and some cancers. In turns, NCDs are the leading cause of sickness and death for women and men, being collectively responsible for almost 70% of all deaths worldwide (https://www.who.int/health-topics/noncommunicable-diseases#tab=tab_1).

Menopause is a critical point for incidence and consequences of obesity and NCDs and, specifically, CVD and T2D. In this way, occurrence and severity of CVD and T2D are lower in premenopausal females than in age-matched males, but dramatically increase after menopause [1,2,3]. The protection against CVD and T2D in women during childbearing age is believed to be related to ovarian estrogens [4,5]. Estrogens prevent visceral adiposity, insulin resistance and glucose intolerance, regulate lipid and free-fatty acids metabolism, increase angiogenesis and vasodilatation, and decrease oxidative stress [5,6,7,8]. Estrogen-based replacement therapies in postmenopausal women reduce the incidence of T2D [9,10], but their role in preventing CVD, and their own security, remains highly controversial [5]. Current data highlights the importance of factors related to the treatment (protocol, route, type of steroids) and, mainly, to the age of the woman and the time of initiation of replacement therapy after menopause [11]. Epidemiological studies indicate that beneficial effects are more frequently found with short-term therapies starting at the early post-menopause [12,13,14], which suggest a very early onset of changes in the metabolism of glucose, lipids and fatty acids.

Therefore, it is necessary to increase the knowledge about the interplay between obesity and menopause and its early effects on women’s health. Interventional research is obviously limited in humans, so preclinical research in animal models is needed. Most of the studies in obesity and menopause have been carried out on laboratory rodents despite the marked differences in metabolism and adipose tissue biology between rodents and humans [15,16]. In this sense, some large animal species offer more profitable characteristics [17]. Among them, pig constitutes a largely known, robust and reliable model for studies on obesity and metabolism, because of omnivorous habits, inclination to sedentarism and metabolic traits similar to humans and mainly because of the high propension for fat deposition of the species [18,19,20,21].

The most commonly used swine breeds for research on obesity and metabolic diseases are Göttingen, Yucatan, and Ossabaw Island pigs. All these breeds have a common ancestor, the Iberian pig, which is characterized by a polymorphism of the leptin receptor gene (LEPR). Leptin and LEPR are involved not only in the regulation of feed intake but also in an ample set of physiological actions and are not only modulated by the energy content of the diet but also by other dietary effects [22]. The LEPR polymorphism of the Iberian pig has been related to effects on food intake, body mass, and fat deposition [23] which resemble leptin resistance in humans [24], but other effects need to be investigated. Iberian pigs, likewise humans, develop obesity, insulin resistance and dyslipidemia in case of food excess and sedentarism during juvenile development [25], adulthood [26], and senescence [27]. Aging and inherent menopause in this model, similarly to humans, are related to sarcopenia and a trend for higher adiposity; in the case of overt obesity, there is increased visceral fattening, dyslipidemia, insulin resistance, lipotoxicity in non-adipose tissues and even obesity-induced renal damage [27,28].

The present study, having in mind these previous considerations about possible metabolic changes during menopause transition and early menopause and about the need of adequate animal models, aimed to characterize a large-animal model based on the use of mature Iberian sows with hormonally-induced menopause. Usually, the estrogen withdrawal associated to menopause is experimentally induced in animal models by surgical ovariectomy [29]. However, abrupt loss of ovarian hormones usually causes more drastic symptoms than spontaneous menopause and, excepting surgical menopause, only about 10% of women cease menstruating abruptly and nearly 90% show a prior period of menstrual abnormality [30]. Moreover, cessation of reproductive competence is primarily driven by the ovarian hormones but there is also a pivotal role of the hypothalamus and hypophysis through changes in the secretion and effects of gonadotrophin-releasing hormone (GnRH) and gonadotrophins FSH and LH [31]. Hence, instead of surgical ovariectomy, we used a treatment based on a GnRH analogue-protein conjugate (Vacsincel^®^, Zoetis, Madrid, Spain) to induce a progressive suppression of the function of the hypothalamus-hypophysis-ovary axis and, thus, of ovarian estrogens secretion.

## 2. Experimental Section

### 2.1. Ethics Statement

The experiment was performed according to the Spanish Policy for Animal Protection RD1201/05, which meets the European Union Directive 86/609, after being specifically assessed and approved by the INIA Committee of Ethics in Animal Research on 11 June 2012 (ethic code CEEA 2012/012). The sows were housed at the animal facilities of the INIA, which meet local, national and European requirements.

### 2.2. Animals and Experimental Procedure

The experiment involved 13 clinically healthy Iberian sows (5–6 years old). All the sows were maintained in two collective pens, one per experimental group, with individual feeders. Animals were fed during the experimental period (120 days) with an obesogenic diet (6.8% of saturated fat) for inducing obesity [26]; the amount was 4 kg/animal/day for the first 30 days, 5 kg/animal/day for the following 60 days and 6 kg/animal/day for the following 30 days Six sows acted as controls (Group CON) whilst seven sows were used as models for menopause (Group MEN). The sows in the group MEN were treated with two doses of Vacsincel^®^ (Zoetis, Madrid, Spain) at Days 0 and 30 of the study, as per manufacturer’s instructions (2 mL/sow injected subcutaneously just behind and below the base of the ear).

In all the sows, body mass and adiposity were measured each 30 days from Day 0 to 120 of the study. Adiposity was evaluated, in terms of subcutaneous back-fat depot at the right-side and at 4 cm from the midline and the head of the last rib, by ultrasonography with a 5–8 MHz lineal-array probe (SonoSite Inc., Bothell, WA, USA). Concomitantly, blood samples were obtained, after around 16 h of fasting, from the orbital sinus using EDTA vacuum tubes (Vacutainer Systems Europe, Becton Dickinson, Meylan Cedex, France). After centrifugation at 1500× *g* for 15 min, aliquots of plasma were separated and stored at −80 °C. The efficiency of the treatment with Vacsincel^®^, and therefore induction of ovarian failure, was assessed by determining plasma progesterone concentrations in one of the plasma aliquots. A second aliquot was used for assessment of plasma parameters of metabolic status. At the end of the experimental period (Day 120), all the sows were sequentially euthanized by stunning and exsanguination, in compliance with RD53/2013 standard procedures. Firstly, the ovaries were examined to confirm data from progesterone assays about ovarian activity, by determining the presence or absence of corpora lutea and follicle development. Immediately, samples of subcutaneous and visceral (perirenal) fat, muscle (longissimus dorsi) and left lobe of the liver were collected and stored at −20 °C for assessment of fatty acids composition.

### 2.3. Assessment of Ovarian Activity

The criteria used to determine the decline of ovarian activity was a decrease in progesterone levels under 2.0 ng/mL. Plasma progesterone concentrations were measured in a single analysis using an enzyme immunoassay kit (Demeditec Diagnostics GmbH, Kiel-Wellsee, Germany), as previously described [32]. Assay sensitivity was 0.045 ng/mL and the intra-assay variation coefficient was 5%. The test was validated for its use in sows by comparing the parallelism of the standard curves produced by standards provided in the assay kit and serially diluted porcine plasma samples with known progesterone concentration [33].

### 2.4. Evaluation of Metabolic Status

The glycemic profile was assessed by determining plasma glucose (GLU) and fructosamine (FRU) concentrations. Lipids metabolism was assessed by determining plasma concentrations of total cholesterol (CHO), high- and low-density lipoproteins cholesterol (HDL-c and LDL-c, respectively), and non-esterified fatty acids (NEFA). Protein metabolism was assessed by determining urea and haptoglobin (HAP). The metabolic state was also assessed through measuring plasma β-hydroxybutyrate (BHB) and lactate (LAC). All metabolites were determined using a clinical chemistry analyzer (Saturno 300 plus, Crony Instruments s.r.l., Rome, Italy) according to manufacturer’s instructions.

### 2.5. Evaluation of Fatty Acids Composition

Fat content and fatty acids composition were determined, as previously described, in samples obtained immediately after euthanasia from adipose (subcutaneous and visceral fat) and non-adipose tissues (muscle and liver). Intramuscular and liver fat were extracted as described by Segura et al. [34] after lyophilization and homogenization; fat content in each tissue was calculated and expressed as a percentage. The neutral lipid fraction (triglycerides) and the polar lipid fraction (phospholipids) were separated using aminopropyl minicolumns previously activated with 7.5 mL of hexane [35]. Subcutaneous and visceral fat were extracted directly; in the case of back-fat, outer and inner layers were analyzed separately, having in mind that the outer layer is more related to thermoregulation whereas the inner layer is more metabolically active [36]. The fatty acids composition of all tissues was analyzed using gas chromatography [37]. The quantities of individual fatty acids expressed as g/100 g of total fatty acid content were used to calculate the proportions of saturated fatty acids (SFA), monounsaturated fatty acids (MUFA), polyunsaturated fatty acids (PUFA), and total omega-3 and omega-6 fatty acids (∑n3 and ∑n6, respectively), as well as the ratios ∑n6/∑n3 and MUFA/SFA and the unsaturation index (UI; calculated as follows: 1 (% monoenoics) + 2 (% dienoics) + 3 (% trienoics) + 4 (% tetraenoics) + 5 (% pentaenoics) + 6 (% hexaenoics)) [38,39]. Furthermore, the desaturation index was used to determine the activity of the stearoyl-CoA desaturase enzyme 1 (SCD1; the ratio of the enzyme product, MUFA mainly oleic acid (C18:1n-9), to the enzyme substrate, SFA mainly stearic acid (C18:0); [40]). Finally, the activities of the desaturase enzymes for n6 and n3 were estimated from the ratios between arachidonic and linoleic acids (C20:4n6/C18:2n6) and eicosapentaenoic and alpha-linolenic acids (C20:5n3/C18:3n3), respectively. ∆9 desaturase activity (D9) was estimated from the ratio between the sum of palmitoleic and oleic acids and the sum of palmitic and stearic acids (C16:1n7 + C18:1n9)/(C16:0 + C18:0).

### 2.6. Statistical Analysis

Effects of ovarian failure on changes in body mass, adiposity, and metabolic status were assessed by ANOVA for repeated measures (split-plot ANOVA). Effects on fatty acids composition were assessed by one-way ANOVA or by a Kruskal–Wallis test if a Levene’s test showed non-homogeneous variables; Duncan’s post-hoc test was performed to contrast the differences among groups. All results were expressed as mean ± S.E.M. Statistical significance was accepted from *p* < 0.05 and a statistical trend was considered when 0.05 < *p* < 0.1.

## 3. Results

**Figure 1:**
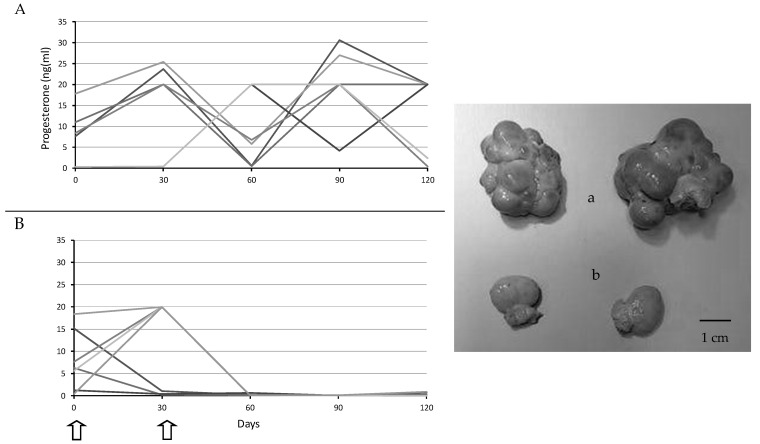
Individual plasma progesterone concentrations over time of study (left hand) and picture of the ovaries at 120 days (right hand) in sows used as controls (**A** and **a**) or treated with two doses of Vacsincel^®^ for inducing ovarian inactivity (**B** and **b**); arrows indicate timing of treatment).

The assessment of plasma progesterone concentrations showed evidence of ovarian cyclic activity throughout the entire period of study in the control sows (Group CON). Conversely, the group treated with Vacsincel^®^ (Group MEN) clearly evidenced ovarian failure and inactivity; from Day 30 in three of the seven females (42.8%) and from Day 60 in the remaining sows. These results were confirmed by the direct observation of the ovaries at Day 120; the ovaries in the Group MEN showed absence of corpora lutea and follicles larger than 2 mm in size and even a strong reduction in the ovarian size (Figure 1).

### 3.1. Changes in Body Mass and Adiposity

**Figure 2:**
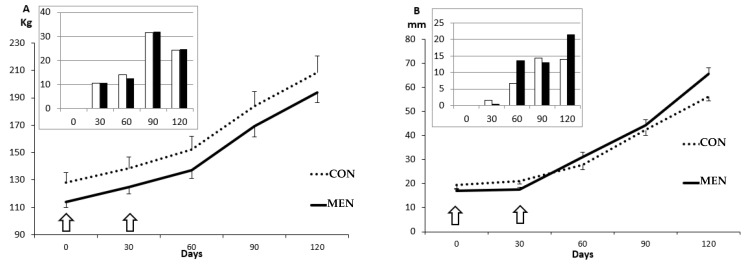

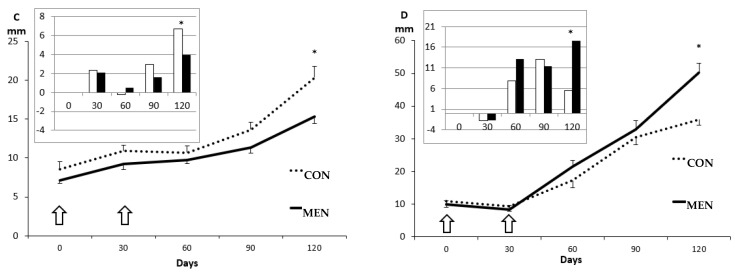
Mean values (± S.E.M.), over time of study, of body mass (**A**) and depth of total (**B**) and outer and inner layers (**C**,**D**) of subcutaneous backfat depots in sows used as controls (discontinuous line) or treated with two doses of Vacsincel^®^ for inducing ovarian inactivity (continuous line; arrows indicate timing of treatment). The inset graphs represent the relative increases when compared to the previous assessment (white group CON, black group MEN); units are the same than in the main graph. Asterisks indicate significant differences between groups (* *p* < 0.05).

All the sows fed with the obesogenic diet, either in the groups CON or MEN, showed a significant increase in body mass (127.0 ± 1.7 to 208.3 ± 2.2 kg for Group CON and 113.9 ± 1.3 to 193.9 ± 1.7 kg for Group MEN; *p* < 0.0001 for both groups) and adiposity (19.5 ± 0.9 to 56.2 ± 1.5 mm of total back-fat depth for Group CON and 17.0 ± 0.6 to 65.6 ± 1.1 mm for Group MEN; *p* < 0.0001 for both groups) from Day 0 to 120 of the study (Figure 2). There were no significant differences between groups in these parameters, either at the beginning or throughout the study. However, we observed significant differences between groups when assessing separately the inner and the outer layers of the subcutaneous back-fat. The sows in the Group MEN showed a trend for a higher deposition at the inner layer and a lower deposition at the outer layer from Day 60 onwards. These differences, reaching statistical significance at Day 120 (*p* < 0.05), were more evident when comparing the relative increases over previous assessments, as depicted in the inset graphs of Figure 2. Conversely, there were no significant differences when comparing the intramuscular fat content (0.20 ± 0.03 to 0.16 ± 0.04%).

### 3.2. Changes in Metabolic Status

**Figure 3:**
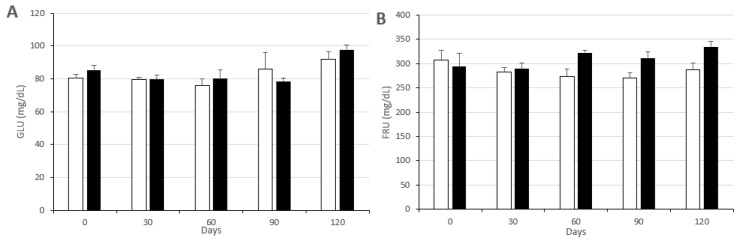

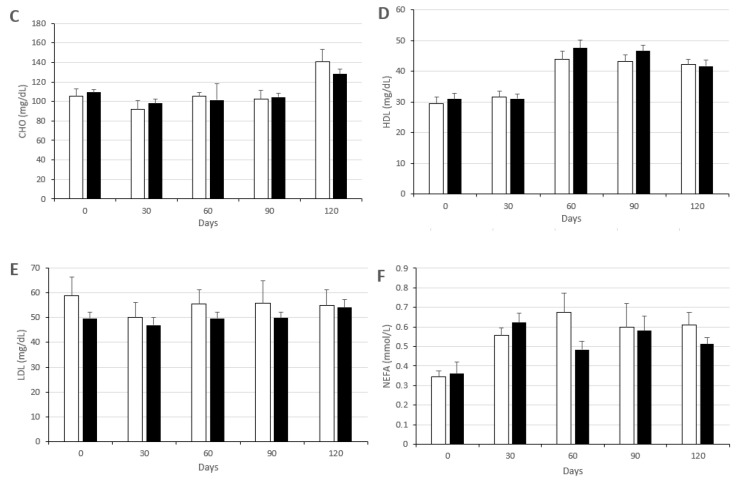
Mean values (± S.E.M.), over time of study, in mean plasma concentrations (± S.E.M.) for glycemic and lipidic metabolic parameters in sows used as controls (group CON; white bars) or treated with two doses of Vacsincel^®^ for inducing ovarian inactivity (group MEN; black bars); GLU: glucose (**A**); FRU: fructosamine (**B**); CHO: total cholesterol (**C**); HDL and LDL: high- and low-density lipoproteins cholesterol, respectively (**D** and **E** respectively); NEFA: non-esterified fatty acids (**F**).

**Figure 4:**
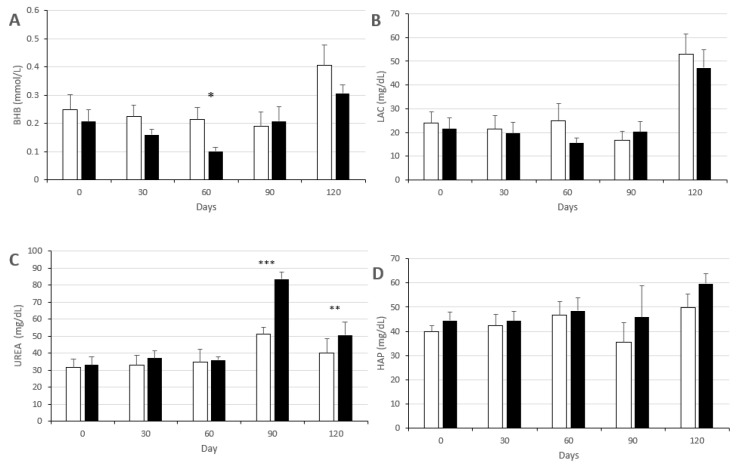
Mean values (± S.E.M.), over time of study, in mean plasma concentrations (± S.E.M.) for metabolic parameters in sows used as controls (group CON; white bars) or treated with two doses of Vacsincel^®^ for inducing ovarian inactivity (group MEN; black bars); BHB: β-hydroxybutyrate (**A**); LAC: lactate (**B**); UREA: urea (**C**) HAP: haptoglobin (**D**). Asterisks indicate significant differences between groups (* *p* < 0.05; ** *p* < 0.01; *** *p* < 0.005).

The intake of a high-fat diet, in both Groups CON and MEN, causes significant increases from Day 0 to 120 of the study (Figure 3) in the mean plasma concentrations of glucose (*p* < 0.05 and *p* < 0.01, respectively), total cholesterol and NEFA (*p* < 0.05 in both groups) and HDL-c (*p* < 0.001 and *p* < 0.005, respectively). Plasma levels of fructosamine increased in both groups between Days 30 and 60, although only significantly in the Group CON (*p* < 0.05) to remain stable in high values until Day 120. Plasma concentrations of urea and lactate (Figure 4) also increased in both groups (*p* < 0.05 in Group CON and *p* < 0.0001 in Group MEN for urea and *p* < 0.05 in both groups for lactate), while haptoglobin increased significantly in the Group MEN (*p* < 0.05).

There were no differences in any of the main metabolic parameters between groups CON and MEN at 0 and 30 days of the study (i.e., prior to completion of the treatment and induction of the ovarian failure in the Group MEN), as depicted in Figure 3 and Figure 4. At 60 days of starting the study, in the first sampling after ovarian hormone stoppage, the sows in the Group MEN showed a trend for lower plasma concentrations of β-hydroxybutyrate (*p* < 0.05) and significantly higher plasma levels of fructosamine (*p* < 0.01). The higher fructosamine levels in the Group MEN were maintained at 90 and 120 days of the study (*p* < 0.05 for both), when plasma urea levels were also significantly higher in the Group MEN (*p* < 0.005 for Day 90 and *p* < 0.01 for Day 120).

### 3.3. Changes in Fatty Acid Composition of Adipose Tissues (Subcutaneous and Visceral Fat)

Most of the significant differences between controls and treated sows (Groups CON and MEN, respectively) were found at the visceral fat and at the inner layer of subcutaneous fat rather than at the outer layer (Table 1 and Tables S1–S3). 

At the visceral fat, contents of myristic (C14:0), palmitic (C16:0), palmitoleic (C16:1n-7) and cis-7 hexadecenoic acids (C16:1n-9) were decreased in the Group MEN when compared to Group CON (*p* < 0.05 for all). There were also found a higher content of total MUFA and oleic acid (C18:1n-9) and a lower proportion of linoleic acid (C18:2n-6) in the Group MEN (*p* = 0.08, *p* < 0.05 and *p* = 0.08, respectively). 

At the subcutaneous fat, the content of palmitoleic acid was also significantly lower at the inner layer of the Group MEN (*p* < 0.05) and the same trend was found at the outer layer (*p* = 0.09); both layers also showed a higher content in docohexaenoic (C22:6n-3; *p* < 0.05 for both) and adrenic acids (C22:4n-6 *p* < 0.05 for the inner and *p* = 0.05 for the outer layer). At the inner layer, the Group MEN also showed trends for lower content of eicosapentaenoic acid (C20:5n-3; *p* = 0.09) and activity of desaturase and elongase enzymes for n3 (DN3; *p* = 0.07), while the outer layer in these sows showed trends for lower content of cis-7 hexadecenoic acid and higher activity of desaturase and elongase enzymes for n6 (DN6; *p* = 0.06 for both). 

### 3.4. Changes in Fatty Acid Composition of Non-Adipose Tissues (Muscle and Liver)

The composition of fatty acids at the intramuscular fat was highly similar between control and treated animals, but the Group MEN evidenced a lower MUFA content at the polar fraction (*p* < 0.05), as detailed in Table 1 and Tables S4 and S5.

Conversely, there were no differences between groups at the polar fraction of the liver whilst the neutral fraction of the liver in the Group MEN showed a higher proportion of docohexaenoic acid (*p* < 0.005), with a trend for higher total n3 content (*p* = 0.09) and lower proportion of eicosapentaenoic and cisvaccenic acids (C18:1n-7; *p* = 0.06 for both), as detailed in Table 1 and Tables S6 and S7.

## 4. Discussion

The results of the present study support previous data on the adequacy and reliability of the Iberian pig fed with a high-fat diet as a translational model of obesity. The sows in the control group of current trial (Group CON) closely reproduced the changes in body mass, adiposity, plasma indexes of carbohydrates and lipids metabolism and fatty acids composition of adipose (subcutaneous and visceral fat) and non-adipose tissues (liver and muscle) previously described for both adult and aged sows of the same breed [26,27]. Current results also support the usefulness of the administration of a GnRH analogue-protein conjugate as an alternative to surgical ovariectomy for inducing ovarian failure and, therefore, for setting up a translational model of menopause. In the present study, the induction of ovarian failure with the use of this compound in sows treated with an obesogenic diet (Group MEN) was related to changes in the patterns of fat deposition, in the fatty acids profiles at the different tissues and in the plasma concentrations of fructosamine, urea, β-hydroxibutirate, and haptoglobin. Altogether, these results indicate that ovarian failure early augments the deleterious effects induced by overfeeding and obesity on metabolic traits and set the basis for future studies on therapeutics of the disease [41].

The abundant intake of the high-fat diet by the sows of the present study, which were 5–6 years-old, caused a daily increase of around 0.7 kg of body mass in both the Group CON and MEN; subcutaneous back-fat depth increased around 0.3 and 0.4 mm/day, respectively. These changes were larger than those found after feeding the same diet to younger sows (2–3 years-old, with 0.4 kg/day and 0.16 mm/day; [26]) and resemble data found in aged sows (8–10 years-old, with 0.8 kg/day and 0.3 mm/day; [27]). A possible weakness of the present study, arising from an experimental point of view, is the lack of a group fed with maintenance diets and no exposed to the obesogenic diet. However, the comparison between sows fed with maintenance and obesogenic diets was broadly performed in these two previous studies and, in agreement with principles of animal welfare in research, we preferred to reduce the number of sows involved in the current study and therefore to focus on changes induced by ovarian failure. 

The assessment of the effects of ovarian stoppage on adiposity indicated that increases in subcutaneous fat during the period of study were around 25% higher in the Group MEN than in the Group CON and were mainly caused by increases at the inner layer (around 37.5% higher in the Group MEN). It is well-known that the inner layer of subcutaneous fat is more metabolically active than the outer layer [36] and even plays a more active role in the regulation of whole-body metabolism than visceral fat [42], in spite that visceral fat is claimed as a higher risk factor for metabolic, immune and inflammatory changes associated to obesity [42,43]. In fact, both the visceral fat and the subcutaneous fat are known to be sensitive to lipolysis and insulin-resistance [44]. However, visceral obesity is more abundant in humans than subcutaneous obesity and, therefore, it is the cause of being commonly related to alterations in lipid profiles and glucose homeostasis [45,46].

Accordingly, most of the significant differences in fatty acids composition between Groups CON and MEN were found at the visceral fat and at the inner layer of the subcutaneous fat. Adipose tissue is the main site for de novo fatty acid synthesis [47], and our previous studies comparing sows fed with maintenance and obesogenic diets [27] showed that a higher adiposity increase MUFA and decrease PUFA contents at both subcutaneous and visceral fat and increase the MUFA/SFA ratio and the desaturation index and Stearoyl-CoA desaturase 1 activity (SCD1) at the visceral fat. The results of the present trial showed a trend for even a higher content of total MUFA and oleic acid (and therefore desaturation index) at the visceral fat of obese sows with stoppage of ovarian hormones (Group MEN). In both human and pigs, increased desaturation index and SCD1 activity have been related to metabolic disorders, like alterations in lipogenesis and insulin regulation [48,49,50,51], in which peroxisome proliferator-activated receptors (PPARs) have a prominent role [52].

On the other hand, oleic acid is protective against insulin resistance [53], because stimulates beta-oxidation of palmitic acid (which was found to be reduced in the visceral fat of Group MEN). At physiological conditions, palmitic acid accumulation is prevented by desaturation to palmitoleic acid and afterwards to oleic acid [54,55]. Hence, oleic acid may reduce palmitic-related insulin resistance and inflammation [56,57,58]. In the current study, palmitic acid and its product palmitoleic acid were found to be decreased at the visceral fat, and also at the inner subcutaneous fat in the case of palmitoleic acid, in the Group MEN. These findings suggest that a protective effect against lipid dysregulation related to these fatty acids [59] is enhanced in the sows of such group. However, we also need to have in mind that an excess of oleic acid induces reactive oxygen species release and mitochondrial dysfunction [60].

We also found changes related to n3 and n6 PUFAs, mainly at the subcutaneous fat of sows in the Group MEN, with higher content of docohexaenoic and eicosapentaenoic acids in spite of a lower activity of enzymes for n-3 (DN3), which convert n6 to n3 fatty acids. Concomitantly, a significantly higher proportion of docohexaenoic acid and a trend for higher total n3 content were found at the neutral fraction of the liver. The docohexaenoic and eicosapentanoic acids are n3 fatty acids which regulate the storage and secretory functions of adipose tissue and have beneficial effects on metabolic and cardiovascular traits [61,62,63]. Previous studies indicate more beneficial effects from docohexaenoic acid [64], which promotes trapping of triglycerides into adipocytes, for improving the systemic metabolic profile. However, an excess of docohexaenoic acid may increase adipogenesis and oxidation of other fatty acids [65,66]. Concomitantly, the increase in adrenic acid found at the inner subcutaneous fat may indicate a dysregulation of the n6 metabolic pathway, which may induce a pro-inflammatory state [67,68].

Altogether, these data suggest that the metabolic state of the Group MEN might be the result of the diverse toxic and protective effects of various fatty acids. In this way, the combined analysis of fatty acids composition and plasma lipids concentrations indicate that regulation of lipids metabolism is affected by obesity but still compensated in early menopause. On the other hand, the assessment of fatty acids composition at the polar fraction of intramuscular fat and plasma indexes for carbohydrates metabolism indicates a higher insulin resistance in the Group MEN.

Skeletal muscle is an essential tissue for whole-body energy metabolism, driving insulin-stimulated glucose uptake [69]. The fatty acid composition at the muscle cellular membranes (i.e.; the polar fraction) has been related to peripheral insulin sensitivity [70,71,72] and, in general, a decreased unsaturation of the fatty acids has been associated with deleterious effects on insulin sensitivity [73] and, finally, with onset of insulin resistance [74]. In current study, the Group MEN evidenced a lower MUFA content which, in agreement with previous considerations, predisposes to insulin resistance. A higher degree of insulin resistance in the Group MEN was supported by assessment of plasma fructosamine concentrations, which were higher than in obese controls from one month after completion of ovarian failure. Plasma fructosamine is indicative of precedent glucose levels, over the previous 2–3 weeks [75], and is therefore considered even more useful than plasma glucose itself for assessing the magnitude of changes in energy balance. Fructosamine results from the irreversible linkage of free aldehyde groups of glucose and the amino group of plasma proteins [76]. Plasma fructosamine therefore depends on average plasma glucose and total protein concentrations, plasma protein composition and rate of plasma protein turnover [75,77]. Hence, plasma fructosamine concentrations mainly increase with prolonged hyperglycemia, although also with decreased protein turnover [78]. 

The data from the current study, showing higher plasma fructosamine concentrations in the Group MEN (i.e., prolonged hyperglycemia), indicate that insulin sensitivity and glucose homeostasis are early affected after ovarian failure. The evidences for alterations in insulin sensitivity found in the present trial are reinforced by significant increases in lactate concentrations over time of study, since such increase is considered a main biomarker of obesity and insulin resistance [79]. However, higher plasma fructosamine concentrations may be also related to decreased protein turnover. Nonetheless, protein turnover includes degradation into urea [80], so decreased protein turnover would be therefore related to lower plasma urea. Conversely, in the present study, the sows of the Group MEN showed increased plasma concentrations of urea from two months after completion of ovarian stoppage. Increases in plasma urea can occur either due to increased urea production or decreased urea elimination through kidney. The design of the present study, unfortunately, does not allow to discern the causes for such high uremia in our sows so it is therefore necessary to develop further specific studies. However, it is important to highlight that men have higher urine urea excretion and serum urea nitrogen levels than women [81] and that blood urea increases in women after menopause [82]. Such findings may indicate a protective effect of estrogens on urea metabolism which, after induction of ovarian failure, was lost in the sows of Group MEN. It is known that the absence of the protective effect of estrogen on the kidneys may contribute to increasing renal disease in postmenopausal women [83]. Hence, having in mind that nutritional management was the same for Groups CON and MEN, we can hypothesize that the renal disorders previously described for our swine model of obesity [28] may be intensified in sows with ovarian hormone stoppage. However, this is also a hypothesis which makes necessary further experimentation.

Finally, when assessing differences in metabolic indexes between Groups CON and MEN, we have to pay attention to the lower concentrations of β-hydroxybutyrate found in the Group MEN. This ketone body is synthesized in the liver from fatty acids to carry energy to peripheral tissues and it is traditionally and widely used as a biomarker of negative energy balance. However, β-hydroxybutyrate is increasingly understood to have cellular signaling functions in metabolic and inflammatory pathways [84] so the decrease found in the Group MEN may be indicative of early dysfunctions at these routes. At the same time, plasma concentrations of haptoglobin are increased at the end of the study in the Group MEN. Haptoglobin is a glycoprotein involved in the acute-phase response to inflammation and its increase in obese subjects seems to be a strong marker of hyperinsulinemia and inflammation [85]. These findings pave the way for further specific research on a possibly increased pro-inflammatory state in the sows with ovarian stoppage. 

## 5. Conclusions

The administration of a GnRH analogue-protein conjugate constitutes a useful method, alternative to surgical ovariectomy, for inducing ovarian stoppage in sows and, therefore, for setting up a translational model of menopause. In the present study, such induction of ovarian failure in sows treated with an obesogenic diet was related to changes in the patterns of fat deposition, in the fatty acids profiles at the different tissues and in the plasma concentrations of fructosamine, urea, β-hydroxibutirate, and haptoglobin when compared to obese sows maintaining ovarian activity. Altogether, these results indicate that menopause early augments the deleterious effects induced by overfeeding and obesity on metabolic traits. However, further studies need to be developed to really point the possible limitations of the model.

## Figures and Tables

**Table 1:** Highlight of significant differences (*p* < 0.05) ± S.E.M. in fatty acid composition in adipose tissue, muscle, and liver of obese sows used as controls or treated with two doses of Vacsincel^®^ for inducing ovarian inactivity (groups CON and MEN, respectively).

Tissue	Layer/Fraction	Variable (g/100 g)	CON	MEN
***SCF***	**Out**	**C16:1n-9**	0.34 ^a^ ± 0.02	0.30 ^b^ ± 0.01
		**C22:4n-6**	0.06 ^a^ ± 0.00	0.07 ^b^ ± 0.00
		**C22:6n-3**	0.01 ^c^ ± 0.00	0.02 ^d^ ± 0.00
		**DN6**	0.01 ^a^ ± 0.00	0.01 ^b^ ± 0.00
	**In**	**C16:1n-7**	2.28 ^c^ ± 0.12	1.87 ^d^ ± 0.12
		**C20:5n-3**	0.01 ^a^ ± 0.00	0.01 ^b^ ± 0.00
		**C22:4n-6**	0.05 ^c^ ± 0.00	0.06 ^d^ ± 0.00
		**C22:6n-3**	0.01 ^c^ ± 0.00	0.02 ^d^ ± 0.00
		**DN3**	0.02 ^a^ ± 0.00	0.02 ^b^ ± 0.00
***VF***		**C14:0**	1.43 ^c^ ± 0.06	1.21 ^d^ ± 0.06
		**C16:0**	28.0 ^c^ ± 0.36	26.8 ^d^ ± 0.45
		**C16:1n-9**	0.38 ^c^ ± 0.02	0.31 ^d^ ± 0.02
		**C16:1n-7**	1.94 ^a^ ± 0.14	1.54 ^b^ ± 0.12
		**C18:1n-9**	36.7 ^c^ ± 0.33	38.4 ^d^ ± 0.54
		**C18:2n-6**	10.7 ^a^ ± 0.41	9.82 ^b^ ± 0.26
		**MUFA**	43.4 ^c^ ± 0.21	44.5 ^d^ ± 0.52
***LD***	**Polar**	**MUFA**	23.5 ^c^ ± 0.20	22.5 ^d^ ± 0.34
***Liver***	**Neutral**	**C18:1n-7**	1.72 ^a^ ± 0.16	2.11 ^b^ ± 0.06
		**C20:5n-3**	0.18 ^a^ ± 0.02	0.14 ^b^ ± 0.01
		**C22:6n-3**	0.85 ^e^ ± 0.08	1.24 ^f^ ± 0.03
		**∑n3**	1.89 ^a^ ± 0.17	2.23 ^b^ ± 0.07

SCF = subcutaneous fat; VF = visceral fat; LD = *longissimus dorsi*; DN6 = total activity of the desaturases of n6; DN3 = total activity of the desaturases of n3; MUFA = sum of monounsaturated FA. Different superscripts indicate significant differences: a≠b *p* < 0.1; c≠d *p* < 0.05; e≠f *p* < 0.005.

## Supplementary Materials

The following are available online at https://www.mdpi.com/2079-7737/9/9/284/s1, Table S1: Differences in fatty acid composition (g/100 g ± S.E.M.) and desaturase activity in the outer layer of the subcutaneous fat between sows used as controls (Group CON) and treated with two doses of Vacsincel^®^ for inducing ovarian inactivity (Group MEN). Table S2: Differences in fatty acid composition (g/100 g ± S.E.M.) and desaturase activity in the inner layer of the subcutaneous fat between sows used as controls (Group CON) and treated with two doses of Vacsincel^®^ for inducing ovarian inactivity (Group MEN). Table S3: Differences in fatty acid composition (g/100 g ± S.E.M.) and desaturase activity in the visceral fat between sows used as controls (Group CON) and treated with two doses of Vacsincel^®^ for inducing ovarian inactivity (Group MEN). Table S4: Differences in fatty acid composition (g/100 g ± S.E.M.) and desaturase activity in the neutral fraction of the longissimus dorsi muscle between sows used as controls (Group CON) and treated with two doses of Vacsincel^®^ for inducing ovarian inactivity (Group MEN). Table S5: Differences in fatty acid composition (g/100 g ± S.E.M.) and desaturase activity in the polar fraction of the longissimus dorsi muscle between sows used as controls (Group CON) and treated with two doses of Vacsincel^®^ for inducing ovarian inactivity (Group MEN). Table S6: Differences in fatty acid composition (g/100 g ± S.E.M.) and desaturase activity in the neutral fraction of the liver between sows used as controls (Group CON) and treated with two doses of Vacsincel^®^ for inducing ovarian inactivity (Group MEN). Table S7: Differences in fatty acid composition (g/100 g ± S.E.M.) and desaturase activity in the polar fraction of the liver between sows used as controls (Group CON) and treated with two doses of Vacsincel^®^ for inducing ovarian inactivity (Group MEN).

## References

[1] Wake R., Yoshiyama M. (2009). Gender differences in ischemic heart disease. Recent Pat. Cardiovasc. Drug Discov..

[2] Hayward C.S., Kelly R.P., Collins P. (2000). The roles of gender, the menopause and hormone replacement on cardiovascular function. Cardiovasc. Res..

[3] Nuutila P., Knuuti M.J., Mäki M., Laine H., Ruotsalainen U., Teräs M., Haaparanta M., Solin O., Yki-Järvinen H. (1995). Gender and insulin sensitivity in the heart and in skeletal muscles. Studies using positron emission tomography. Diabetes.

[4] Cignarella A., Bolego C. (2010). Mechanisms of estrogen protection in diabetes and metabolic disease. Horm. Mol. Biol. Clin. Investig..

[5] Iorga A., Cunningham C.M., Moazeni S., Ruffenach G., Umar S., Eghbali M. (2017). The protective role of estrogen and estrogen receptors in cardiovascular disease and the controversial use of estrogen therapy. Biol. Sex Differ..

[6] Louet J.F., LeMay C., Mauvais-Jarvis F. (2004). Antidiabetic actions of estrogen: Insight from human and genetic mouse models. Curr. Atheroscler. Rep..

[7] Mauvais-Jarvis F., Clegg D.J., Hevener A.L. (2013). The role of estrogens in control of energy balance and glucose homeostasis. Endocr. Rev..

[8] Rhee Y., Paik M.J., Kim K.R., Ko Y.G., Kang E.S., Cha B.S., Lee H.C., Lim S.K. (2008). Plasma free fatty acid level patterns according to cardiovascular risk status in postmenopausal women. Clin. Chim. Acta.

[9] Kanaya A.M., Herrington D., Vittinghoff E., Lin F., Grady D., Bittner V., Cauley J.A., Barrett-Connor E. (2003). Heart and estrogen/progestin replacement study. Glycemic effects of postmenopausal hormone therapy: The heart and estrogen/progestin replacement study: A randomized, double-blind, placebo-controlled trial. Ann. Intern. Med..

[10] Margolis K.L., Bonds D.E., Rodabough R.J., Tinker L., Phillips L.S., Allen C., Bassford T., Burke G., Torrens J., Howard B.V. (2004). Effect of oestrogen plus progestin on the incidence of diabetes in postmenopausal women: Results from the Women’s Health Initiative Hormone Trial. Diabetologia.

[11] Yang X.P., Reckelhoff J.F. (2011). Estrogen, hormonal replacement therapy and cardiovascular disease. Curr. Opin. Nephrol. Hypertens..

[12] Schierbeck L.L., Rejnmark L., Tofteng C.L., Stilgren L., Eiken P., Mosekilde L., Køber L., Jensen J.E. (2012). Effect of hormone replacement therapy on cardiovascular events in recently postmenopausal women: Randomised trial. BMJ.

[13] Wolff E.F., He Y., Black D.M., Brinton E.A., Budoff M.J., Cedars M.I., Hodis H.N., Lobo R.A., Manson J.E., Merriam G.R. (2013). Self-reported menopausal symptoms, coronary artery calcification, and carotid intima-media thickness in recently menopausal women screened for the Kronos early estrogen prevention study (KEEPS). Fertil. Steril..

[14] De Villiers T.J., Hall J.E., Pinkerton J.V., Cerdas Pérez S., Rees M., Yang C., Pierroz D.D. (2016). Revised global consensus statement on menopausal hormone therapy. Climacteric.

[15] Arner P. (2005). Resistin: Yet another adipokine tells us that men are not mice. Diabetologia.

[16] Russell J.C., Proctor S.D. (2006). Small animal models of cardiovascular disease: Tools for the study of the roles of metabolic syndrome, dyslipidemia, and atherosclerosis. Cardiovasc. Pathol..

[17] Hamernik D.L. (2019). Farm animals are important biomedical models. Anim. Front..

[18] Lunney J.K. (2007). Advances in swine biomedical model genomics. Int. J. Biol. Sci..

[19] Douglas W.R. (1972). Of pigs and men and research: A review of applications and analogies of the pig, sus scrofa, in human medical research. Space Life Sci..

[20] Spurlock M.E., Gabler N.K. (2008). The development of porcine models of obesity and the metabolic syndrome. J. Nutr..

[21] LittenBrown J.C., Corson A.M., Clarke L. (2010). Porcine models for the metabolic syndrome, digestive and bone disorders: A general overview. Animal.

[22] Cappai M.G., Dall’Aglio C., Sander S.J., Ratert C., Dimauro C., Pinna W., Kamphues J. (2016). Different physical forms of one diet fed to growing pigs induce morphological changes in mandubular glands and local leptin (Ob) production and receptor (ObR) expression. J. Anim. Physiol. Anim. Nutr..

[23] Ovilo C., Fernández A., Noguera J.L., Barragán C., Letón R., Rodríguez C., Mercadé A., Alves E., Folch J.M., Varona L. (2005). Fine mapping of porcine chromosome 6 QTL and LEPR effects on body composition inmultiple generations of an iberian by landrace intercross. Genet. Res..

[24] Myers M.G., Cowley M.A., Munzberg H. (2008). Mechanisms of leptin action and leptin resistance. Annu. Rev. Physiol..

[25] Torres-Rovira L., Gonzalez-Anover P., Astiz S., Caro A., Lopez-Bote C., Ovilo C., Pallares P., Perez-Solana M.L., Sanchez-Sanchez R., Gonzalez-Bulnes A. (2013). Effect of an obesogenic diet during the juvenile period on growth pattern, fatness and metabolic, cardiovascular and reproductive features of swine with obesity/leptin resistance. Endocr. Metab. Immune Disord. Drug Targets.

[26] Torres-Rovira L., Astiz S., Caro A., Lopez-Bote C., Ovilo C., Pallares P., Perez-Solana M.L., Sanchez-Sanchez R., Gonzalez-Bulnes A. (2012). Diet-induced swine model with obesity/leptin resistance for the study of metabolic syndrome and type 2 diabetes. Sci. World J..

[27] Garcia-Contreras C., Vazquez-Gomez M., Torres-Rovira L., Gonzalez J., Porrini E., Gonzalez-Colaço M., Isabel B., Astiz S., Gonzalez-Bulnes A. (2018). Characterization of ageing- and diet-related swine models of sarcopenia and sarcopenic obesity. Int. J. Mol. Sci..

[28] Rodríguez-Rodríguez R., González-Bulnes A., Garcia-Contreras C., Rodriguez-Rodriguez A.E., Astiz S., Vazquez-Gomez M., Pesantez J.L., Isabel B., Salido-Ruiz E., González J. (2020). The Iberian pig fed with high-fat diet: A model of renal disease in obesity and metabolic syndrome. Int. J. Obes..

[29] Chalvon-Demersay T., Blachier F., Tomé D., Blais A. (2017). Animal models for the study of the relationships between diet and obesity: A focus on dietary protein and estrogen deficiency. Front. Nutr..

[30] Kim W.W. (2017). Endocrinology of the Menopause First Consensus Meeting on Menopause in the East Asian Region. https://www.gfmer.ch/Books/bookmp/33.htm.

[31] Hall J.E. (2015). Endocrinology of the menopause. Endocrinol. Metab. Clin. N. Am..

[32] Ueshiba H., Zerah M., New M.I. (1994). Enzyme-linked immunosorbent assay (ELISA). Method for screening of nonclassical steroid 21-hydroxylase deficiency. Norm. Metab. Res..

[33] Gonzalez-Añover P., Encinas T., Gomez-Izquierdo E., Sanz E., Letelier C.A., Torres-Rovira L., Pallares P., Sanchez-Sanchez R., Gonzalez-Bulnes A. (2010). Advanced onset of puberty in gilts of thrifty genotype (Iberian pig). Reprod. Dom. Anim..

[34] Segura J., Lopez-Bote C.J. (2014). A laboratory efficient method for intramuscular fat analysis. Food Chem..

[35] Ruiz J., Antequera T., Andres A.I., Petron M.J., Muriel E. (2004). Improvement of a solid phase extraction method for analysis of lipid fractions in muscle foods. Anal. Chim. Acta.

[36] Daza A., Latorre M.A., Olivares A., López Bote C.J. (2016). The effects of male and female immunocastration on growth performances and carcass and meat quality of pigs intended for dry-cured ham production: A preliminary study. Livest. Sci..

[37] Lopez-Bote C., Rey A., Ruiz J., Isabel B., Sanz Arias R. (1997). Effect of feeding diets high in monounsaturated fatty acids and α-tocopheryl acetate to rabbits on resulting carcass fatty acid profile and lipid oxidation. Anim. Sci..

[38] Segura J., Escudero R., Romero de Avila M.D., Cambero M.I., Lopez-Bote C.J. (2015). Effect of fatty acid composition and positional distribution within the triglyceride on selected physical properties of dry-cured ham subcutaneous fat. Meat Sci..

[39] Hulbert A.J., Pamplona R., Buffenstein R., Buttemer W.A. (2007). Life and death: Metabolic rate, membrane composition, and life span of animals. Physiol. Rev..

[40] Hulver M.W., Berggren J.R., Carper M.J., Miyazaki M., Ntambi J.M., Hoffman E.P., Thyfault J.P., Stevens R., Dohm G.L., Houmard J.A. (2005). Elevated stearoyl-CoA desaturase-1 expression in skeletal muscle contributes to abnormal fatty acid partitioning in obese humans. Cell Metab..

[41] Caruso S., Cianci S., Cariola M., Fava V., Rapisarda M.C., Cianci A. (2017). Effects of nutraceuticals on quality of life and sexual function of perimenopausal women. J. Endocrinol. Investig..

[42] Zhou C., Zhang J., Ma J., Jiang A., Tang G., Mai M., Zhu L., Bai L., Li M., Li X. (2013). Gene expression profiling reveals distinct features of various porcine adipose tissues. Lipids Health Dis..

[43] Wronska A., Kmiec Z. (2012). Structural and biochemical characteristics of various white adipose tissue depots. Acta Physiol..

[44] Ibrahim M.M. (2010). Subcutaneous and visceral adipose tissue: Structural and functional differences. Obes. Rev..

[45] Després J.P. (2001). Health consequences of visceral obesity. Ann. Med..

[46] Lafontan M., Girard J. (2008). Impact of visceral adipose tissue on liver metabolism. Part I: Heterogeneity of adipose tissue and functional properties of visceral adipose tissue. Diabetes Metab..

[47] Bergen W.G., Mersmann H.J. (2005). Comparative aspects of lipid metabolism: Impact on contemporary research and use of animal models. J. Nutr..

[48] Roden M., Price T.B., Perseghin G., Petersen K.F., Rothman D.L., Cline G.W., Shulman G.I. (1996). Mechanism of free fatty acid-induced insulin resistance in humans. J. Clin. Investig..

[49] Poudyal H., Brown L. (2011). Stearoyl-CoA desaturase: A vital checkpoint in the development and progression of obesity. Endocr. Metab. Immune Disord. Drug Targets.

[50] Barbero A., Astiz S., Lopez-Bote C.J., Perez-Solana M.L., Ayuso M., Garcia-Real I., Gonzalez-Bulnes A. (2013). Maternal malnutrition and offspring sex determine juvenile obesity and metabolic disorders in a swine model of leptin resistance. PLoS ONE.

[51] Gonzalez-Bulnes A., Astiz S., Ovilo C., Lopez-Bote C.J., Sanchez-Sanchez R., Perez-Solana M.L., Torres-Rovira L., Ayuso M., Gonzalez J. (2014). Early-postnatal changes in adiposity and lipids profile by transgenerational developmental programming in swine with obesity/leptin resistance. J. Endocrinol..

[52] Laganà A.S., Vitale S.G., Nigro A., Sofo V., Salmeri F.M., Rossetti P., Rapisarda A.M., La Vignera S., Condorelli R.A., Rizzo G. (2016). Pleiotropic actions of peroxisome proliferator-activated receptors (PPARs) in dysregulated metabolic homeostasis, inflammation and cancer: Current evidence and future perspectives. Int. J. Mol. Sci..

[53] Tardif N., Salles J., Landrier J.F., Mothe-Satney I., Guillet C., BoueVaysse C., Combaret L., Giraudet C., Patrac V., Bertrand-Michel J. (2011). Oleate enriched diet improves insulin sensitivity and restores muscle protein synthesis in old rats. Clin. Nutr..

[54] Strable M.S., Ntambi J.M. (2010). Genetic control of de novo lipogenesis: Role in diet-induced obesity. Crit. Rev. Biochem. Mol. Biol..

[55] Silbernagel G., Kovarova M., Cegan A., Machann J., Schick F., Lehmann R., Häring H.U., Stefan N., Schleicher E., Fritsche A. (2012). High hepatic SCD1 activity is associated with low liver fat content in healthy subjects under a lipogenic diet. J. Clin. Endocrinol. Metab..

[56] Coll T., Eyre E., Rodríguez-Calvo R., Palomer X., Sánchez R.M., Merlos M., Laguna J.C., Vázquez-Carrera M. (2008). Oleate reverses palmitate-induced insulin resistance and inflammation in skeletal muscle cells. J. Biol. Chem..

[57] Gao D., Griffiths H.R., Bailey C.J. (2009). Oleate protects against palmitate-induced insulin resistance in L6 myotubes. Br. J. Nutr..

[58] Martínez-García C., Izquierdo-Lahuerta A., Vivas Y., Velasco I., Yeo T.K., Chen S., Medina-Gomez G. (2015). Renal lipotoxicity-associated inflammation and insulin resistance affects actin cytoskeleton organization in podocytes. PLoS ONE.

[59] Kang M., Lee A., Yoo H.J., Kim M., Kim M., Shin D.Y., Lee J.H. (2017). Association between increased visceral fat area and alterations in plasma fatty acid profile in overweight subjects: A crosssectional study. Lipids Health Dis..

[60] Arany I., Clark J., Reed D., Juncos L., Dixit M. (2013). The role of p66shc in renal toxicity of oleic acid. Am. J. Nephrol..

[61] Puglisi M.J., Hasty A.H., Saraswathi V. (2011). The role of adipose tissue in mediating the beneficial effects of dietary fish oil. J. Nutr. Biochem..

[62] Carpentier Y.A., Portois L., Malaisse W.J. (2006). N-3 fatty acids and the metabolic syndrome. Am. J. Clin. Nutr..

[63] Mozaffarian D., Wu J.H. (2012). (n − 3) Fatty acids and cardiovascular health: Are effects of EPA and DHA shared or complementary?. J. Nutr..

[64] Wei M.Y., Jacobson T.A. (2011). Effects of eicosapentaenoic acid versus docosahexaenoic acid on serum lipids: A systematic review and meta-analysis. Curr. Atheroscler. Rep..

[65] Huang C.W., Chen Y.J., Yang J.T., Chen C.Y., Ajuwon K.M., Chen S.E., Su N.W., Chen Y.S., Mersmann H.J., Ding S.T. (2017). Docosahexaenoic acid increases accumulation of adipocyte triacylglycerol through up-regulation of lipogenic gene expression in pigs. Lipids Health Dis..

[66] Murali G., Desouza C.V., Clevenger M.E., Ramalingam R., Saraswathi V. (2014). Differential effects of eicosapentaenoic acid and docosahexaenoic acid in promoting the differentiation of 3 T3-L1 preadipocytes. Prostaglandins Leukot. Essent. Fat. Acids.

[67] Del Cornò M., D’Archivio M., Conti L., Scazzocchio B., Varì R., Donninelli G., Varano B., Giammarioli S., De Meo S., Silecchia G. (2016). Visceral fat adipocytes from obese and colorectal cancer subjects exhibit distinct secretory and ω6 polyunsaturated fatty acid profiles and deliver immunosuppressive signals to innate immunity cells. Oncotarget.

[68] Horas H., Nababan S., Nishiumi S., Kawano Y., Kobayashi T., Yoshida M., Azuma T. (2017). Adrenic acid as an inflammation enhancer in non-alcoholic fatty liver disease. Arch. Biochem. Biophys..

[69] DeFronzo R.A., Jacot E., Jequier E., Maeder E., Wahren J., Felber J.P. (1981). The effect of insulin on the disposal of intravenous glucose. Results from indirect calorimetry and hepatic and femoral venous catheterization. Diabetes.

[70] Borkman M., Storlien L.H., Pan D.A., Jenkins A.B., Chisholm D.J., Campbell L.V. (1993). The relation between insulin sensitivity and the fatty acid composition of skeletal-muscle phospholipids. N. Engl. J. Med..

[71] Clore J.N., Li J., Gill R., Gupta S., Spencer R., Azzam A., Zuelzer W., Rizzo W.B., Blackard W.G. (1998). Skeletal muscle phosphatidylcholine fatty acids and insulin sensitivity in normal humans. Am. J. Physiol. Endocrinol. Metab..

[72] Pan D.A., Lillioja S., Milner M.R., Kriketos A.D., Baur L.A., Bogardus C., Storlien L.H. (1995). Skeletal muscle membrane lipid composition is related to adiposity and insulin action. J. Clin. Investig..

[73] Storlien L.H., Pan D.A., Kriketos A.D., O’Connor J., Caterson I.D., Cooney G.J., Jenkins A.B., Baur L.A. (1996). Skeletal muscle membrane lipids and insulin resistance. Lipids.

[74] Li Y., Xu S., Zhang X., Yi Z., Cichello S. (2015). Skeletal intramyocellular lipid metabolism and insulin resistance. Biophys. Rep..

[75] Pattullo K.M., Kidney B.A. (2014). Exploring fructosamine beyond diabetes mellitus. J. Am. Vet. Med. Assoc..

[76] Armbruster D.A. (1987). Fructosamine: Structure, analysis and clinical usefulness. Clin. Chem..

[77] Mosca A., Carenini A., Zoppi F., Carpinelli A., Banfi G., Ceriotti F., Bonini P., Pozza G. (1987). Plasma protein glycation as measured by fructosamine assay. Clin. Chem..

[78] Bernstein R.E. (1987). Nonenzymatically glycated proteins. Adv. Clin. Chem..

[79] Lovejoy J., Newby F.D., Gebhart S.S., DiGirolamo M. (1992). Insulin resistance in obesity is associated with elevated basal lactate levels and diminished lactate appearance following intravenous glucose and insulin. Metabolism.

[80] Schutz Y. (2011). Protein turnover, ureagenesis and gluconeogenesis. Int. J. Vitam. Nutr. Res..

[81] Chang A.R., Zafar W., Grams M.E. (2018). Kidney function in obesity-challenges in indexing and estimation. Adv. Chronic Kidney Dis..

[82] Curry A.S. (2018). Biochemistry of Women: Clinical Concepts.

[83] Atapattu P.M. (2015). Obesity at menopause: An expanding problem. J. Pat. Care.

[84] Newman J.C., Verdin E. (2017). β-hydroxybutyrate: A signaling metabolite. Annu. Rev. Nutr..

[85] De Pergola G., Di Roma P., Paoli G., Guida P., Pannacciulli N., Giorgino R. (2007). Haptoglobin serum levels are independently associated with insulinemia in overweight and obese women. J. Endocrinol. Investig..

