# Supplementary material for: Short-Term Effects of Early Menopause on Adiposity, Fatty Acids Profile and Insulin Sensitivity of a Swine Model of Female Obesity
Source: Biology (Basel). 2020 Sep 11;9(9):284. doi: 10.3390/biology9090284 (PMC7565410; doi:10.3390/biology9090284)
Supplement: Supplementary file 1 [file biology-09-00284-s001.pdf]

biology

MDPI

Article

Short-Term Effects of Early Menopause on Adiposity, Fatty Acids Profile and Insulin Sensitivity of a Swine Model of Female Obesity

Ana Heras-Molina, José Luis Pesantez-Pacheco, Marta Vazquez-Gomez, Consolacion Garcia-Contreras, Susana Astiz, Beatriz Isabel and Antonio Gonzalez-Bulnes

Table S1. Differences in fatty acid composition (g/100 g ± S.E.M.) and desaturase activity in the outer layer of the subcutaneous fat between sows used as controls (Group CON) and treated with two doses of Vacsincel® for inducing ovarian inactivity (Group MEN).

Variable	CON	MEN	*P*-value
C14:0	1.22 ± 0.04	1.13 ± 0.04	0.11
C16:0	22.2 ± 0.40	22.1 ± 0.34	0.77
C16:1n-9	0.34 ± 0.02	0.30 ± 0.01	0.06
C16:1n-7	2.50 ± 0.16	2.11 ± 0.14	0.09
C17:0	0.28 ± 0.02	0.30 ± 0.03	0.60
C17:1	0.34 ± 0.03	0.31 ± 0.03	0.48
C18:0	9.07 ± 0.55	10.2 ± 0.62	0.19
C18:1n-9	46.6 ± 0.38	47.0 ± 0.57	0.60
C18:1n-7	4.34 ± 0.39	3.97 ± 0.26	0.43
C18:2n-6	10.6 ± 0.33	10.0 ± 0.23	0.15
C18:3n-6	0.01 ± 0.00	0.01 ± 0.00	0.96
C18:3n-3	0.59 ± 0.04	0.55 ± 0.03	0.50
C18:4n-3	0.07 ± 0.01	0.07 ± 0.01	0.88
C20:1n-9	1.41 ± 0.07	1.57 ± 0.07	0.12
C20:3n-6	0.09 ± 0.01	0.07 ± 0.01	0.13
C20:4n-6	0.10 ± 0.01	0.11 ± 0.01	0.28
C20:5n-3	0.02 ± 0.00	0.01 ± 0.00	0.73
C22:4n-6	0.06 ± 0.00	0.07 ± 0.00	0.05
C22:5n-3	0.05 ± 0.00	0.05 ± 0.00	0.47
C22:6n-3	0.01 ± 0.00	0.02 ± 0.00	0.02
SFA	32.8 ± 0.86	33.8 ± 0.89	0.47
MUFA	55.6 ± 0.64	55.3 ± 0.67	0.76
PUFA	11.6 ± 0.37	11.0 ± 0.26	0.17
MUFA/SFA	1.70 ± 0.06	1.65 ± 0.07	0.58
UI	0.80 ± 0.01	0.79 ± 0.01	0.37
∑n3	0.73 ± 0.05	0.70 ± 0.04	0.62
∑n6	10.9 ± 0.35	10.3 ± 0.23	0.16
∑n3/∑n6	15.1 ± 0.80	14.8 ± 0.54	0.73
D9	0.61 ± 0.01	0.60 ± 0.01	0.54
DI	5.74 ± 0.40	5.11 ± 0.36	0.26
DN3	0.03 ± 0.01	0.02 ± 0.01	0.75
DN6	0.01 ± 0.00	0.01 ± 0.00	0.06

SFA = sum of saturated fatty acids; MUFA = sum o monounsaturated fatty acids; PUFA = sum of polyunsaturated fatty acids; UI = unsaturation index; D9 = activity of Δ9 desaturase activity; DI = desaturation index; DN3 total activity of the desaturases of n3; DN6 = total activity of the desaturases of n6.

Table 2. Differences in fatty acid composition (g/100 g ± S.E.M.) and desaturase activity in the inner layer of the subcutaneous fat between sows used as controls (Group CON) and treated with two doses of Vacsincel® for inducing ovarian inactivity (Group MEN).

**Variable**	**CON**	**MEN**	***P*-value**
C14:0	1.33 ± 0.06	1.25 ± 0.05	0.31
C16:0	24.4 ± 0.48	24.0 ± 0.34	0.50
C16:1n-9	0.26 ± 0.02	0.23 ± 0.01	0.20
C16:1n-7	2.28 ± 0.12	1.87 ± 0.12	0.03
C17:0	0.28 ± 0.02	0.30 ± 0.03	0.49
C17:1	0.28 ± 0.03	0.26 ± 0.03	0.69
C18:0	11.7 ± 0.69	13.0 ± 0.70	0.21
C18:1n-9	43.7 ± 0.77	43.8 ± 0.54	0.93
C18:1n-7	4.15 ± 0.41	3.93 ± 0.41	0.72
C18:2n-6	9.59 ± 0.44	9.24 ± 0.18	0.46
C18:3n-6	0.01 ± 0.00	0.01 ± 0.00	0.50
C18:3n-3	0.54 ± 0.04	0.53 ± 0.04	0.93
C18:4n-3	0.05 ± 0.00	0.05 ± 0.00	0.91
C20:1n-9	1.13 ± 0.05	1.25 ± 0.05	0.12
C20:3n-6	0.06 ± 0.00	0.07 ± 0.01	0.46
C20:4n-6	0.08 ± 0.00	0.08 ± 0.00	0.97
C20:5n-3	0.01 ± 0.00	0.01 ± 0.00	0.09
C22:4n-6	0.05 ± 0.00	0.06 ± 0.00	0.01
C22:5n-3	0.13 ± 0.09	0.05 ± 0.01	0.35
C22:6n-3	0.01 ± 0.00	0.02 ± 0.01	0.04
SFA	37.7 ± 1.15	38.6 ± 0.92	0.57
MUFA	51.8 ± 1.12	51.3 ± 0.73	0.73
PUFA	10.5 ± 0.48	10.1 ± 0.21	0.43
MUFA/SFA	1.38 ± 0.07	1.34 ± 0.05	0.60
UI	0.74 ± 0.01	0.73 ± 0.01	0.44
∑n3	0.74 ± 0.11	0.66 ± 0.04	0.50
∑n6	9.79 ± 0.45	9.46 ± 0.19	0.49
∑n3/∑n6	14.4 ± 1.51	14.6 ± 0.70	0.91
D9	0.56 ± 0.01	0.55 ± 0.01	0.59
DI	4.20 ± 0.32	3.75 ± 0.24	0.28
DN3	0.02 ± 0.00	0.02 ± 0.00	0.07
DN6	0.01 ± 0.00	0.01 ± 0.00	0.68

SFA = saturated fatty acids; MUFA = sum o monounsaturated fatty acids; PUFA = sum of polyunsaturated fatty acids; UI = unsaturation index; D9 = activity of Δ9 desaturase activity; DI = desaturation index; DN3 total activity of the desaturases of n3; DN6 = total activity of the desaturases of n6.

Table S3. Differences in fatty acid composition (g/100 g ± S.E.M.) and desaturase activity in the visceral fat between sows used as controls (Group CON) and treated with two doses of Vacsincel® for inducing ovarian inactivity (Group MEN).

**Variable**	**CON**	**MEN**	***P*-value**
C14:0	1.43 ± 0.06	1.21 ± 0.06	0.02
C16:0	28.0 ± 0.36	26.6 ± 0.45	0.03
C16:1n-9	0.38 ± 0.02	0.31 ± 0.02	0.04
C16:1n-7	1.94 ± 0.14	1.54 ± 0.12	0.04
C17:0	0.34 ± 0.03	0.39 ± 0.03	0.27
C17:1	0.23 ± 0.02	0.24 ± 0.02	0.73
C18:0	15.1 ± 0.58	16.5 ± 0.62	0.14
C18:1n-9	36.7 ± 0.33	38.5 ± 0.54	0.03
C18:1n-7	3.31 ± 0.11	3.13 ± 0.13	0.30
C18:2n-6	10.7 ± 0.41	9.82 ± 0.26	0.08
C18:3n-6	0.02 ± 0.00	0.02 ± 0.00	0.11
C18:3n-3	0.61 ± 0 03	0.60 ± 0.03	0.72
C18:4n-3	0.04 ± 0.00	0.04 ± 0.00	0.32
C20:1n-9	0.76 ± 0.02	0.85 ± 0.07	0.25
C20:3n-6	0.10 ± 0.01	0.11 ± 0.01	0.63
C20:4n-6	0.08 ± 0.01	0.08 ± 0.01	0.82

C20:5n-3	0.01 ± 0.00	0.01 ± 0.00	0.13
C22:4n-6	0.05 ± 0.01	0.06 ± 0.00	0.69
C22:5n-3	0.06 ± 0.01	0.06 ± 0.01	0.77
C22:6n-3	0.02 ± 0.00	0.02 ± 0.00	0.21
SFA	44.9 ± 0.53	44.7 ± 0.74	0.79
MUFA	43.4 ± 0.21	44.5 ± 0.52	0.08
PUFA	11.7 ± 0.45	10.8 ± 0.30	0.11
MUFA/SFA	0.97 ± 0.02	1.00 ± 0.03	0.35
UI	0.68 ± 0.01	0.68 ± 0.01	0.67
∑n3	0.74 ± 0.03	0.73 ± 0.03	0.77
∑n6	11.0 ± 0.42	10.1 ± 0.27	0.10
∑n3/∑n6	14.9 ± 0.53	14.0 ± 0.41	0.19
D9	0.47 ± 0.00	0.48 ± 0.01	0.32
DI	2.66 ± 0.09	2.54 ± 0.10	0.39
DN3	0.02 ± 0.00	0.02 ± 0.00	0.15
DN6	0.01 ± 0.00	0.01 ± 0.00	0.64

SFA = saturated fatty acids; MUFA = sum o monounsaturated fatty acids; PUFA = sum of polyunsaturated fatty acids; UI = unsaturation index; D9 = activity of Δ9 desaturase activity; DI = desaturation index; DN3 total activity of the desaturases of n3; DN6 = total activity of the desaturases of n6.

Table S4. Differences in fatty acid composition (g/100 g ± S.E.M.) and desaturase activity in the neutral fraction of the longissimus dorsi muscle between sows used as controls (Group CON) and treated with two doses of Vacsincel® for inducing ovarian inactivity (Group MEN).

**Variable**	**CON**	**MEN**	***P*-value**
C14:0	1.34 ± 0.04	1.25 ± 0.05	0.16
C16:0	24.3 ±0.65	23.7 ± 0.44	0.45
C16:1n-9	0.30 ± 0.01	0.33 ± 0.02	0.24
C16:1n-7	4.82 ±0.26	4.36 ± 0.37	0.35
C17:0	0.17 ± 0.01	0.18 ± 0.01	0.39
C17:1	0.24 ± 0.02	0.25 ± 0.01	0.68
C18:0	10.0 ± 0.68	9.95 ± 0.51	0.91
C18:1n-9	4.35 ± 0.34	4.27 ± 0.31	0.87
C18:1n-7	47.6 ± 0.52	48.5 ± 0.44	0.21
C18:2n-6	5.08 ± 0.32	5.33 ± 0.31	0.59
C18:3n-6	0.03 ± 0.00	0.03 ± 0.00	0.13
C18:3n-3	0.38 ± 0.04	0.44 ± 0.04	0.27
C18:4n-3	0.09 ± 0.01	0.10 ± 0.01	0.31
C20:1n-9	0.89 ± 0.04	0.94 ± 0.07	0.56
C20:3n-6	0.05 ± 0.01	0.06 ± 0.00	0.67
C20:4n-6	0.16 ± 0.02	0.16 ± 0.01	0.92
C20:5n-3	0.01 ± 0.00	0.01 ± 0.00	0.69
C22:4n-6	0.07 ± 0.01	0.07 ± 0.00	0.80
C22:5n-3	0.05 ± 0.01	0.06 ± 0.01	0.73
C22:6n-3	0.03 ± 0.00	0.03 ± 0.00	0.60
SFA	35.9 ± 1.27	35.1 ± 0.82	0.61
MUFA	58.2 ± 1.04	58.6 ± 0.74	0.73
PUFA	5.95 ± 0.36	6.29 ± 0.35	0.52
MUFA/SFA	1.64 ± 0.09	1.68 ± 0.06	0.70
UI	0.71 ± 0.02	0.73 ± 0.01	0.54
∑n3	0.57 ± 0.04	0.65 ± 0.04	0.21
∑n6	5.39 ± 0.34	5.64 ± 0.32	0.60
∑n3/∑n6	9.58 ± 0.60	8.81 ± 0.40	0.30
D9	0.21 ± 0.02	0.20 ± 0.01	0.72
DI	5.31 ± 0.42	5.41 ± 0.35	0.86
DN3	0.04 ± 0.01	0.03 ± 0.00	0.24
DN6	0.03 ± 0.00	0.03 ± 0.00	0.70

SFA = saturated fatty acids; MUFA = sum o monounsaturated fatty acids; PUFA = sum of polyunsaturated fatty acids; UI = unsaturation index; D9 = activity of Δ9 desaturase activity; DI = desaturation index; DN3 total activity of the desaturases of n3; DN6 = total activity of the desaturases of n6.

Table S5. Differences in fatty acid composition (g/100 g ± S.E.M.) and desaturase activity in the polar fraction of the longissimus dorsi muscle between sows used as controls (Group CON) and treated with two doses of Vacsincel® for inducing ovarian inactivity (Group MEN).

Variable	CON	MEN	*P*-value
C14:0	3.84 ± 0.58	4.23 ± 0.19	0.52
C16:0	28.7 ± 1.47	28.1 ± 0.64	0.69
C16:1n-9	0.66 ± 0.12	0.71 ± 0.04	0.69
C16:1n-7	1.14 ± 0.11	1.13 ± 0.04	0.91
C17:0	0.67 ± 0.04	0.67 ± 0.05	0.91
C17:1	1.07 ± 0.05	1.15 ± 0.11	0.55
C18:0	10.2 ± 0.90	9.27 ± 0.60	0.39
C18:1n-9	15.5 ± 0.32	14.7 ± 0.41	0.14
C18:1n-7	4.49 ± 0.24	4.22 ± 0.15	0.36
C18:2n-6	25.2 ± 1.30	26.5 ± 0.61	0.35
C18:3n-6	0.17 ± 0.01	0.20 ± 0.01	0.13
C18:3n-3	0.43 ± 0.02	0.40 ± 0.02	0.28
C18:4n-3	0.04 ± 0.01	0.06 ± 0.01	0.27
C20:1n-9	0.63 ± 0.07	0.60 ± 0.05	0.77
C20:3n-6	0.44 ± 0.05	0.49 ± 0.02	0.38
C20:4n-6	4.63 ± 0.49	5.31 ± 0.35	0.27
C20:5n-3	0.66 ± 0.09	0.72 ± 0.04	0.55
C22:4n-6	0.48 ± 0.06	0.54 ± 0.04	0.43
C22:5n-3	0.48 ± 0.06	0.50 ± 0.05	0.88
C22:6n-3	0.58 ± 0.03	0.59 ± 0.04	0.91
SFA	43.4 ± 1.74	42.2 ± 0.84	0.54
MUFA	23.5 ± 0.20	22.5 ± 0.34	0.03
PUFA	33.1 ± 1.83	35.3 ± 0.96	0.29
MUFA/SFA	0.55 ± 0.02	0.53 ± 0.01	0.60
UI	1.07 ± 0.05	1.12 ± 0.02	0.34
∑n3	2.20 ± 0.12	2.26 ± 0.04	0.60
∑n6	30.9 ± 1.73	33.0 ± 0.94	0.28
∑n3/∑n6	14.1 ± 0.41	14.6 ± 0.40	0.38
D9	0.30 ± 0.01	0.30 ± 0.01	0.71
DI	2.03 ± 0.16	2.08 ± 0.12	0.79
DN3	1.52 ± 0.22	1.81 ± 0.13	0.26
DN6	0.18 ± 0.01	0.20 ± 0.01	0.38

SFA = saturated fatty acids; MUFA = sum o monounsaturated fatty acids; PUFA = sum of polyunsaturated fatty acids; UI = unsaturation index; D9 = activity of Δ9 desaturase activity; DI = desaturation index; DN3 total activity of the desaturases of n3; DN6 = total activity of the desaturases of n6.

Table S6. Differences in fatty acid composition (g/100 g ± S.E.M.) and desaturase activity in the neutral fraction of the liver between sows used as controls (Group CON) and treated with two doses of Vacsincel® for inducing ovarian inactivity (Group MEN).

Variable	CON	MEN	*P*-value
C14:0	0.54 ± 0.09	0.60 ± 0.03	0.63
C16:0	22.6 ± 0.91	23.8 ± 0.34	0.31
C16:1n-9	0.38 ± 0.07	0.43 ± 0.03	0.49
C16:1n-7	0.93 ± 0.07	0.89 ± 0.03	0.73
C17:0	2.29 ± 0.42	1.68 ± 0.16	0.16
C17:1	0.61 ± 0.13	0.49 ± 0.05	0.34
C18:0	35.3 ± 1.71	34.5 ± 0.65	0.75
C18:1n-9	17.3 ± 1.10	16.8 ± 0.41	0.66

C18:1n-7	1.72 ± 0.16	2.11 ± 0.06	0.06
C18:2n-6	9.34 ± 1.17	8.59 ± 0.44	0.55
C18:3n-6	0.14 ± 0.02	0.12 ± 0.01	0.63
C18:3n-3	0.25 ± 0.04	0.22 ± 0.01	0.43
C18:4n-3	0.15 ± 0.05	0.15 ± 0.02	0.90
C20:1n-9	0.85 ± 0.12	0.88 ± 0.05	0.82
C20:3n-6	0.71 ± 0.19	0.52 ± 0.07	0.31
C20:4n-6	5.15 ± 0.57	5.97 ± 0.22	0.41
C20:5n-3	0.18 ± 0.02	0.14 ± 0.01	0.06
C22:4n-6	0.29 ± 0.05	0.37 ± 0.02	0.26
C22:5n-3	0.45 ± 0.08	0.49 ± 0.03	0.70
C22:6n-3	0.85 ± 0.08	1.24 ± 0.03	0.00
SFA	60.7 ± 1.76	60.6 ± 0.66	0.98
MUFA	21.8 ± 1.25	21.6 ± 0.47	0.88
PUFA	17.5 ± 1.85	17.8 ± 0.70	0.90
MUFA/SFA	0.36 ± 0.03	0.36 ± 0.01	0.90
UI	0.74 ± 0.05	0.78 ± 0.02	0.59
∑n3	1.89 ± 0.17	2.23 ± 0.07	0.09
∑n6	15.6 ± 1.80	15.6 ± 0.68	0.98
∑n3/∑n6	8.49 ± 1.16	6.98 ± 0.44	0.23
D9	0.24 ± 0.01	0.23 ± 0.00	0.66
DI	0.55 ± 0.05	0.56 ± 0.02	0.92
DN3	0.82 ± 0.14	0.66 ± 0.05	0.32
DN6	0.56 ± 0.04	0.69 ± 0.02	0.14

SFA = saturated fatty acids; MUFA = sum o monounsaturated fatty acids; PUFA = sum of polyunsaturated fatty acids; UI = unsaturation index; D9 = activity of Δ9 desaturase activity; DI = desaturation index; DN3 total activity of the desaturases of n3; DN6 = total activity of the desaturases of n6.

Table S7. Differences in fatty acid composition (g/100 g ± S.E.M.) and desaturase activity in the polar fraction of the liver between sows used as controls (Group CON) and treated with two doses of Vacsincel® for inducing ovarian inactivity (Group MEN).

**Variable**	**CON**	**MEN**	***P*-value**
C14:0	0.31 ± 0.03	0.33 ± 0.04	0.73
C16:0	23.8 ± 0.93	23.6 ± 1.27	0.90
C16:1n-9	0.26 ± 0.01	0.27 ± 0.01	0.56
C16:1n-7	0.84 ± 0.10	0.86 ± 0.08	0.87
C17:0	1.56 ± 0.21	1.30 ± 0.18	0.36
C17:1	0.36 ± 0.06	0.30 ± 0.03	0.37
C18:0	30.5 ± 1.00	29.6 ± 1.02	0.54
C18:1n-9	18.0 ± 0.64	18.0 ± 0.87	0.97
C18:1n-7	1.58 ± 0.17	1.52 ± 0.11	0.79
C18:2n-6	12.8 ± 0.80	12.6 ± 0.71	0.89
C18:3n-6	0.11 ± 0.02	0.09 ± 0.01	0.36
C18:3n-3	0.15 ± 0.03	0.15 ± 0.02	0.81
C18:4n-3	0.02 ± 0.00	0.02 ± 0.00	0.71
C20:1n-9	0.52 ± 0.08	0.37 ± 0.05	0.11
C20:3n-6	0.74 ± 0.18	0.61 ± 0.08	0.50
C20:4n-6	6.43 ± 0.48	8.03 ± 1.14	0.25
C20:5n-3	0.21 ± 0.01	0.18 ± 0.02	0.33
C22:4n-6	0.27 ± 0.02	0.39 ± 0.07	0.16
C22:5n-3	0.70 ± 0.07	0.79 ± 0.13	0.57
C22:6n-3	0.90 ± 0.06	1.04 ± 0.06	0.11
SFA	56.2 ± 0.95	54.8 ± 1.37	0.45
MUFA	21.6 ± 0.70	21.3 ± 1.00	0.82
PUFA	22.3 ± 1.07	23.9± 1.72	0.45
MUFA/SFA	0.38 ± 0.02	0.39 ± 0.02	0.85
UI	0.87 ± 0.02	0.94 ± 0.05	0.29

∑n3	1.97 ± 0.05	2.19 ± 0.12	0.15
∑n6	20.3 ± 1.08	21.7 ± 1.61	0.49
∑n3/∑n6	10.4 ± 0.67	9.90 ± 0.31	0.50
D9	0.26 ± 0.01	0.26 ± 0.01	0.81
DI	0.65 ± 0.03	0.67 ± 0.05	0.74
DN3	1.59 ± 0.23	1.30 ± 0.23	0.40
DN6	0.51 ± 0.04	0.64 ± 0.08	0.22

SFA = saturated fatty acids; MUFA = sum o monounsaturated fatty acids; PUFA = sum of polyunsaturated fatty acids; UI = unsaturation index; D9 = activity of Δ9 desaturase activity; DI = desaturation index; DN3 total activity of the desaturases of n3; DN6 = total activity of the desaturases of n6.

© 2020 by the authors. Licensee MDPI, Basel, Switzerland. This article is an open access article distributed under the terms and conditions of the Creative Commons Attribution (CC BY) license (http://creativecommons.org/licenses/by/4.0/).
